# Automatic evaluation of atlantoaxial subluxation in rheumatoid arthritis by a deep learning model

**DOI:** 10.1186/s13075-023-03172-x

**Published:** 2023-09-25

**Authors:** Yasutaka Okita, Toru Hirano, Bowen Wang, Yuta Nakashima, Saki Minoda, Hajime Nagahara, Atsushi Kumanogoh

**Affiliations:** 1 Department of Respiratory Medicine and Clinical Immunology, Osaka University Graduate School of Medicine, 2-2, Yamadaoka, Suita, Osaka 565-0871 Japan; 2 Department of Rheumatology, Nishinomiya Municipal Central Hospital, Hyogo, Japan; 3 Osaka University Institute for Datability Science (IDS), Suita, Osaka Japan; 4 Laboratory of Immunopathology, World Premier International Immunology Frontier Research Center, Osaka University, Suita, Osaka Japan; 5 The Institute for Open and Transdisciplinary Research Initiatives (OTRI), Osaka, Japan

**Keywords:** Rheumatoid arthritis, Deep learning, Machine learning, Cervical spine, Atlantoaxial subluxation

## Abstract

**Background:**

This work aims to develop a deep learning model, assessing atlantoaxial subluxation (AAS) in rheumatoid arthritis (RA), which can often be ambiguous in clinical practice.

**Methods:**

We collected 4691 X-ray images of the cervical spine of the 906 patients with RA. Among these images, 3480 were used for training the deep learning model, 803 were used for validating the model during the training process, and the remaining 408 were used for testing the performance of the trained model. The two-dimensional key points’ detection model of Deep High-Resolution Representation Learning for Human Pose Estimation was adopted as the base convolutional neural network model. The model inferred four coordinates to calculate the atlantodental interval (ADI) and space available for the spinal cord (SAC). Finally, these values were compared with those by clinicians to evaluate the performance of the model.

**Results:**

Among the 408 cervical images for testing the performance, the trained model correctly identified the four coordinates in 99.5% of the dataset. The values of ADI and SAC were positively correlated among the model and two clinicians. The sensitivity of AAS diagnosis with ADI or SAC by the model was 0.86 and 0.97 respectively. The specificity of that was 0.57 and 0.5 respectively.

**Conclusions:**

We present the development of a deep learning model for the evaluation of cervical lesions of patients with RA. The model was demonstrably shown to be useful for quantitative evaluation.

**Supplementary Information:**

The online version contains supplementary material available at 10.1186/s13075-023-03172-x.

## Background

Rheumatoid arthritis (RA) is a chronic autoimmune disease characterized by synovitis and multiple joint destruction. The spine is affected in addition to the joints of extremities in patients with RA. Cervical spine lesion is the most frequent spinal lesion due to RA and it is found in 25–80% of patients with RA [1–10]. The frequency of cervical spine lesions in patients with RA is not necessarily decreasing [11, 12], even though many patients achieve remission or low disease activity [13]. One of the reasons is that the patients with RA have been aging [14] and the cervical spine lesions are also affected by aging. Cervical spine lesions are classified into two types, upper and middle/lower cervical spine lesions, with upper cervical spine lesions being more common [12]. Cervical spine lesion causes not only neck pain but also vascular compression [15], radiculopathy, and myelopathy because of cervical spine instability, which can be life-threatening [16]. Thus, patients with cervical spine lesions require careful attention in the management of RA. Screening and reproducible evaluation by X-ray examination of atlantoaxial subluxation (AAS) of the upper cervical spine are important, because it is the most common type [17] and a risk for complications of other types of cervical lesions such as vertical subluxation of the axis and subaxial subluxation [5]. AAS is diagnosed with an atlantodental interval (ADI) of more than 3 mm or the space available for the spinal cord (SAC) of 13 mm or less [18]. However, the cervical subluxation is often overlooked for several reasons. Firstly, half of patients with radiographic instability are asymptomatic [3]. Secondly, typical neurological abnormalities such as hyperreflexia are not observed due to peripheral joint destruction. Thirdly, radiographic evidence of AAS sometimes appears to be decreased in X-ray images due to the development of basilar invagination (pseudostabilization) [11].

Recently, artificial intelligence (AI) has been developed and applied in medicine such as the diagnosis of diabetic retinopathy [19] and support for endoscopic diagnosis [20]. Convolutional neural network (CNN), which is an algorithm suitable for image processing among AI, shows remarkable performance in classifying various images, including the diagnosis of skin cancer [21, 22], the detection of lymph node metastases in women with breast cancer [23], classification of interstitial lung diseases [24], and analysis of genomic data [25]. As an additional merit, AI can process large amounts of data in a short time with good reproducibility. In the field of RA, CNNs have been developed for several uses, such as the diagnosis of RA [26], and evaluation of synovitis by ultrasound images [27, 28]. Previously, we developed CNN for the analysis of finger joints by X-ray imaging, which is the gold standard for the evaluation of joint destruction due to RA [29].

In this work, we developed a deep learning model to evaluate AAS on cervical spine X-ray images of patients with RA and evaluated its performance. The identification of four coordinates is mandatory for ADI and SAC calculations. The two-dimensional key points’ detection model of Deep High-Resolution Representation Learning for Human Pose Estimation (HRNet) [30] was adopted as the base CNN model.

## Methods

### Patients and images

Nine hundred and thirty-six patients diagnosed with RA based on the American Rheumatism Association 1987 revised criteria [31] or the 2010 ACR-EULAR RA classification criteria [32] were enrolled. All patients received treatment at Osaka University Hospital and were enrolled in the institute’s cohort of patients with RA. Clinical information, such as age, gender, and disease duration, was collected from medical records. The present work was approved by the Ethics Committee of Osaka University Hospital and was conducted according to the Declaration of Helsinki. The board waived the requirement for patient informed consent by posting the opt-out information on the hospital’s home page.

We retrospectively collected digital X-ray images of neutral position of the right lateral cervical spine and anteflexion/retroflexion positions if they had been taken, that were taken from January 1, 2016, to May 31, 2021. The images of 21 patients were excluded because of a history of cervical spine surgery as well as those of nine additional patients because of extremely severe destruction of the cervical spine, in which the atlantoaxial joint could not be identified. Finally, 4691 images of the 906 patients were used for machine learning.

The nine hundred and six patients were given serial numbers and the numbers were randomly sorted, by numpy.ramdom.permutation function in NumPy 1.21.6. The patients from the beginning to the 680th in the sequence were selected for the training dataset, the patients from the 681st to 820th were selected for the training dataset, and the remaining patients were selected for the test dataset. Finally, a training dataset to adjust network parameters included 3480 images of 680 patients, a validation dataset to monitor the training process included 803 images of 140 patients, and a test dataset to assess the trained model included 408 images of 86 patients.

### Machine learning step

Based on the consensus of two rheumatologists (Y.O. and S.M.), all images were labeled with four coordinates essential for ADI and SAC calculation and Digital Imaging and Communications in Medicine (DICOM) tag (0028,0030) meaning pixel spacing (length per pixel). The machine learning program was written using Python 3.6.9, NumPy 1.24.3, OpenCV-Python 4.1.2.30, and Matplotlib 3.2.2. The two-dimensional key points’ detection model of HRNet which is able to maintain high-resolution representations through the whole process and give great performance was adopted as the base CNN model. It was combined with various modules (transfer learning and ADI and SAC calculations) to improve performance. The batch size for training was 32.

### Model performance evaluation

Using the test dataset, we examined the performance of the trained model by comparing the ADI and SAC assessed by the model to those by the two clinicians. Sensitivity and specificity were calculated when the judgment of clinician 1 was defined as true; that is, an ADI ≥ 3 mm or SAC ≤ 13 mm was determined as AAS positive. Differences in ADI and SAC among the model, clinician 1, and clinician 2 were compared and statistically examined using an equivalence test with a hypothesis value of 0 and a range of 1.5 mm for ADI or 2 mm for SAC. Statistical analyses were conducted using JMP software (Pro 16.0.0).

## Results

### Patients and images

Table 1 shows the characteristics of the 906 patients. The proportion of females was 77.9%. The median and interquartile range of age and disease duration at the time the first X-ray image was obtained were 66 years (54–73 years) and 8 years (1–17 years), respectively. The seropositivity of rheumatoid factor was 79.0% and that of anti-citrullinated peptide antibody was 63.0%. The profiles of patients indicated that the participants enrolled in this work were representative of the general RA population. The values of ADI and SAC measured by clinicians are shown in sFig. 1. The median and interquartile range of ADI and SAC were 1.61 mm (1.00–2.92 mm) and 19.81 mm (17.23–21.52 mm), respectively.

**Table 1:** Characteristics of the patients

Characteristic	Total
*n*	906
Sex, female/male	706 / 200
Age, years, median (interquartile ranges)	66 (54, 73)
Disease duration, years, median (interquartile ranges)	8 (1, 17)
RF positive, %	79.0%
ACPA positive, %	63.0%
Number of radiographs	4691

*RF* rheumatoid factor, *ACPA* anti-citrullinated peptide antibody

### Key points’ detection

**Fig. 1:**
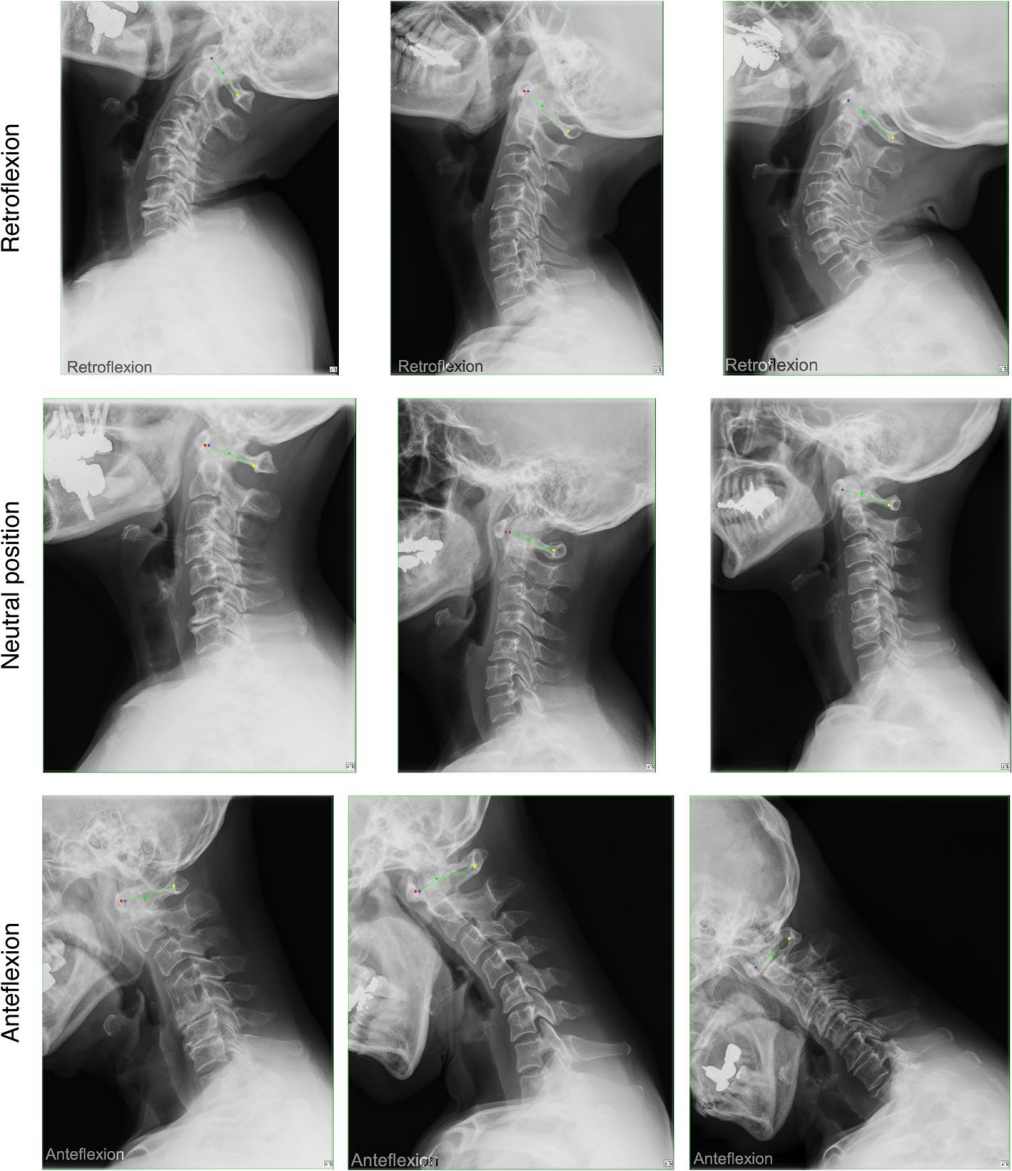
Detection of four coordinates for atlantodental interval and space available for the spinal cord. Representative images processed by the model. The four coordinates mandatory for atlantodental interval and space available for the spinal cord calculations detected by the model are shown in red, purple, green, and yellow dots from the ventral side

As shown in Fig. 1a, the four coordinates detected by the model were shown in red, purple, green, and yellow dots from the ventral side. The heatmap loss value and Percentage of Correct Keypoints (PCK) were less than 0.0001 and 0.2551 at 40 epochs, respectively. The mean distance between true and estimated coordinates is 2.66 mm (standard deviation 4.98 mm). The distance was more than 10 mm in two images, suggesting an estimated probability of 99.5%. These two images were judged to be outliers.

### Evaluation of model performance

**Fig. 2:**
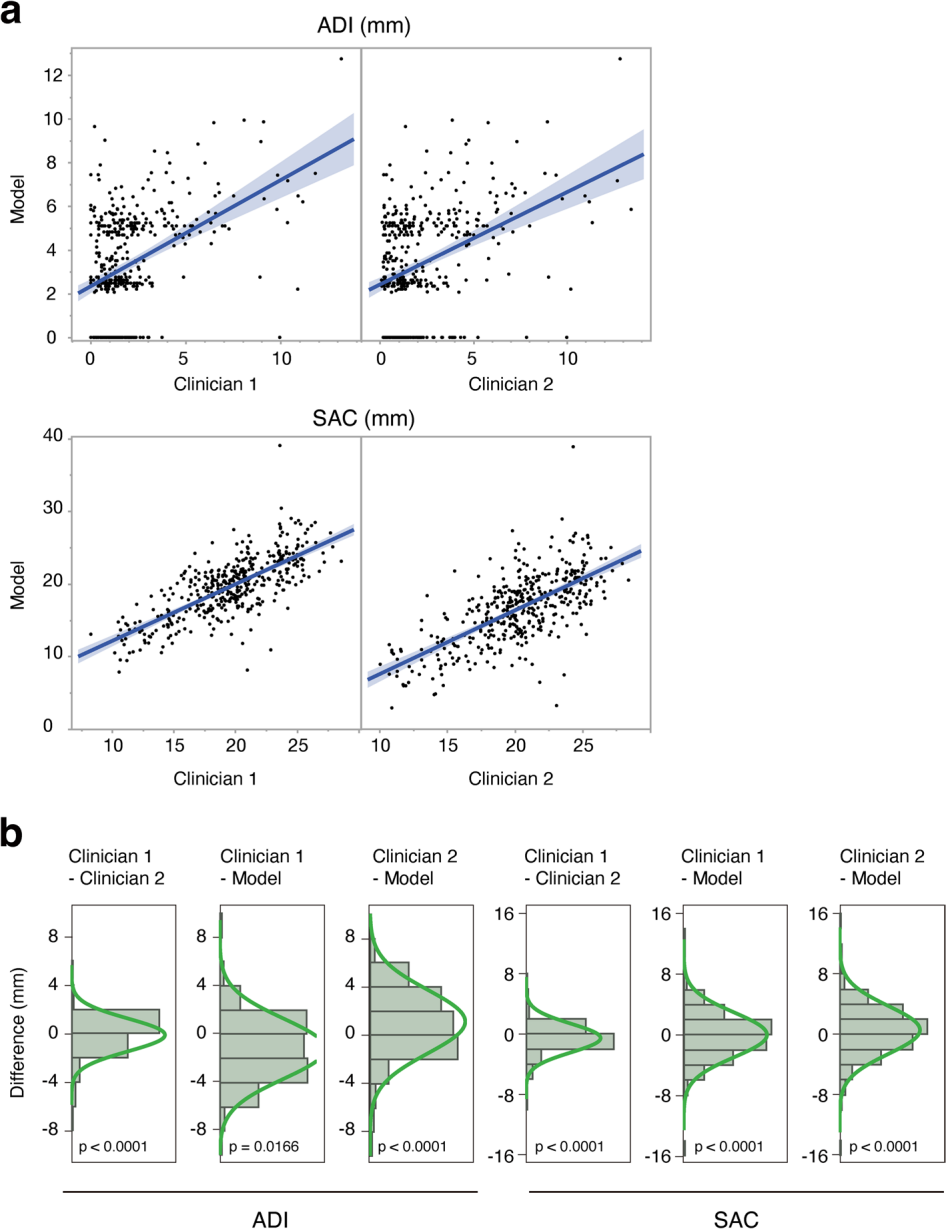
Evaluation of model performance. **a** Correlations of values of atlantodental interval (ADI, upper column) or space available for the spinal cord (SAC, lower column) among the model, clinician 1, and clinician 2. **b** Distribution of the difference of  ADI (left three columns) and  SAC (right three columns) among the model, clinician 1, and clinician 2. Each graph is scaled by the range of ADI or  SAC. The values were statistically examined by equivalence test with hypothesis value zero with a range of ± 1.5 mm for ADI and ± 2.0 mm for SAC

As shown in Fig. 2a, the values of ADI were positively correlated among the model, clinician 1, and clinician 2 (the correlation coefficients were 0.43 and 0.40 respectively). The values of SAC were also positively correlated among the model, clinician 1, and clinician 2 (the correlation coefficients were 0.73 and 0.68 respectively). The differences in ADI or SAC among the model, clinician 1, and clinician 2 are shown in Fig. 2b. These differences were normally distributed and could be interpreted as zero with a range of ± 1.5 mm for ADI and ± 2.0 mm for SAC by equivalence test.

The sensitivity of AAS diagnosis with ADI by the model was 0.86 and the specificity was 0.57 (Table 2). The sensitivity of AAS diagnosis with SAC by the model was 0.97 and the specificity was 0.5 (Table 2).

**Table 2:** Diagnosis of atlantoaxial subluxation

ADI		By the model	
		≥ 3 mm	< 3 mm
By clinician 1	≥ 3 mm	68	11
	< 3 mm	141	186
By clinician 2	≥ 3 mm	83	23
	< 3 mm	126	174
SAC		By the model	
		≥ 13 mm	< 13 mm
By clinician 1	≥ 13 mm	367	11
	< 13 mm	14	14
By clinician 2	≥ 13 mm	372	14
	< 13 mm	9	11

*ADI* atlantodental interval, *SAC* space available for the spinal cord

## Discussion

We developed a deep learning model for atlantoaxial joint detection and ADI/SAC evaluation, using images of the cervical spine from patients with RA. While developing the machine learning model, we created and evaluated several algorithms such as programs to formulate the key points’ detection as a CNN-based regression problems. Consequently, this model which is able to maintain high-resolution representations through the whole process and gave the best performance was reported.

In daily clinical practice, it is often difficult to diagnose cervical spine lesions for several reasons. Half of patients with cervical spine lesions are asymptomatic [3]. Deep tendon reflexes are not often exaggerated because of limb joint destruction in patients with RA. There are some cases where radiographic evidence of AAS sometimes appear to be decreased in X-ray image due to the development of basilar invagination. This pattern has been described as “pseudostabilization.” Therefore, cervical spine lesions can be discovered at advanced stage such as the Ranawat classification Class IIIB [33, 34]. Our model could diagnose AAS with a sensitivity of 0.97 and specificity of 0.57. Our findings show that the developed AI-based model can accurately and rapidly identify AAS, thereby potentially reducing oversight of AAS in patients in clinical settings.

Cervical spine lesion in RA is progressive, and spinal cord symptoms may result in quadriplegia or shortened lifespan [35]. Thus, it is necessary to evaluate disease progression over time using a reliable approach to ensure timely treatment for the suppression of cervical spine destruction. In addition to identifying AAS, our model can be also useful in assessing the interval change of cervical spine lesions in patients with RA over time as the outputs of ADI and SAC are numerical values.

Our work has a few limitations. First, X-ray images used in this study were retrospectively collected. Prospective studies are needed to verify whether our model is really useful in assessing changes in cervical spine lesions in RA patients over time. Second, X-ray images used in this study were taken at Osaka University Hospital. Further machine learning with images taken at other hospitals is required to improve the validity and robustness of the model. Third, our model can assess AAS, but it cannot assess vertical subluxation of the axis or subaxial subluxation, both of which are also found in patients with RA. As a next step, it is considered necessary to develop AI to evaluate them. Fourth, our model could not detect the four coordinates in two images. In one of them, the patient bent forward until almost horizontal.

## Conclusions

We introduced a deep learning model using CNN to assess atlantoaxial joint destruction in patients with RA. This model provides a partial assessment of the many joints that can be destroyed in RA. The introduction of AI in healthcare can be useful for oversight prevention, time-savings, reductions in effort, health surveys, and assessments by healthcare professionals, both specialists and non-specialists. Importantly, our methodology can be applied to other joints, such as the elbow, shoulder, hip, knee, foot, and spine, as well as for other conditions or disorders, including osteoporosis, fracture, and bone tumor. We conclude that image processing with a trained CNN model is a promising tool to assess radiographic cervical destruction in RA.

### Supplementary Information

**Additional file 1: Sup Fig. 1.** Distribution of ADI and SAC. **a** Atlantodental interval (ADI, mm). **b** Space available for the spinal cord (SAC, mm).

## Data Availability

The datasets used and analyzed during the current work are available from the corresponding author upon reasonable request. The code is available at https://github.com/ya-o/neck_RA.

## Abbreviations

AAS: Atlantoaxial subluxation
ADI: Atlantodental interval
AI: Artificial intelligence
CNN: Convolutional neural network
RA: Rheumatoid arthritis
SAC: Space available for the spinal cord
