# Supplementary figures and images for: Automatic evaluation of atlantoaxial subluxation in rheumatoid arthritis by a deep learning model
Source: Arthritis Res Ther. 2023 Sep 25;25:181. doi: 10.1186/s13075-023-03172-x (PMC10518918; doi:10.1186/s13075-023-03172-x)

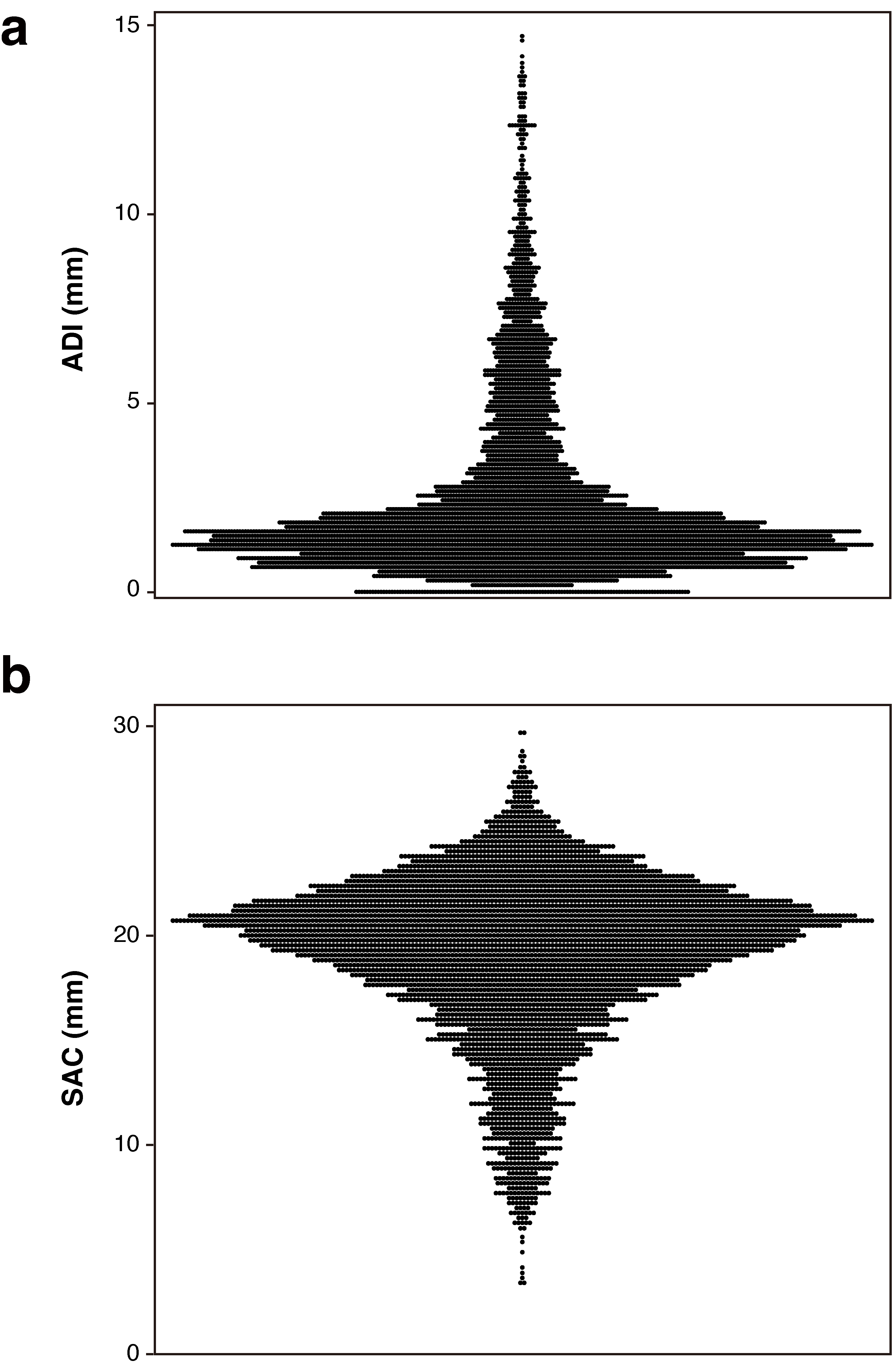

Supplement: Supplementary file 1 — Additional file 1: Sup Fig. 1. Distribution of ADI and SAC. a Atlantodental interval (ADI, mm). b Space available for the spinal cord (SAC, mm). [file 13075_2023_3172_MOESM1_ESM.tif]
